# Supplementary material for: Multi‐dataset identification of innovative feature genes and molecular mechanisms in keratoconus
Source: J Cell Mol Med. 2024 Sep 19;28(18):e70079. doi: 10.1111/jcmm.70079 (PMC11412914; doi:10.1111/jcmm.70079)
Supplement: Supplementary file 2 — Material S2. [file JCMM-28-e70079-s002.zip › 7.GSEA analysis/3.DBNDD1/KEGG_PRION_DISEASES.html]

Details for gene set KEGG\_PRION\_DISEASES[GSEA]

|  || Dataset | DBNDD1.group.cls#h\_versus\_l |
| Phenotype | group.cls#h\_versus\_l |
| Upregulated in class | l |
| GeneSet | KEGG\_PRION\_DISEASES |
| Enrichment Score (ES) | -0.41429827 |
| Normalized Enrichment Score (NES) | -1.1814488 |
| Nominal p-value | 0.22874494 |
| FDR q-value | 0.70288336 |
| FWER p-Value | 0.991 |
Table: GSEA Results Summary

  

Fig 1: Enrichment plot: KEGG\_PRION\_DISEASES      
 Profile of the Running ES Score & Positions of GeneSet Members on the Rank Ordered List

  

| SYMBOL | TITLE | RANK IN GENE LIST | RANK METRIC SCORE | RUNNING ES | CORE ENRICHMENT || 1 | ELK1 | na | 3923 | 0.216 | -0.0536 | No |
| 2 | C8A | na | 5449 | 0.166 | -0.0519 | No |
| 3 | C6 | na | 8447 | 0.096 | -0.1120 | No |
| 4 | MAPK3 | na | 9865 | 0.073 | -0.1326 | No |
| 5 | C1QA | na | 10386 | 0.066 | -0.1295 | No |
| 6 | MAP2K2 | na | 10931 | 0.059 | -0.1289 | No |
| 7 | HSPA1A | na | 13025 | 0.038 | -0.1787 | No |
| 8 | C9 | na | 13377 | 0.035 | -0.1792 | No |
| 9 | BAX | na | 13486 | 0.034 | -0.1728 | No |
| 10 | C1QC | na | 14542 | 0.026 | -0.1960 | No |
| 11 | PRKX | na | 15799 | 0.017 | -0.2275 | No |
| 12 | NOTCH1 | na | 16241 | 0.014 | -0.2363 | No |
| 13 | C8B | na | 17744 | 0.005 | -0.2783 | No |
| 14 | C7 | na | 19181 | -0.006 | -0.3180 | No |
| 15 | STIP1 | na | 20365 | -0.016 | -0.3477 | No |
| 16 | PRKACA | na | 20814 | -0.020 | -0.3550 | No |
| 17 | SOD1 | na | 20880 | -0.021 | -0.3511 | No |
| 18 | MAPK1 | na | 22705 | -0.040 | -0.3927 | No |
| 19 | EGR1 | na | 22708 | -0.040 | -0.3818 | No |
| 20 | C8G | na | 23047 | -0.044 | -0.3795 | No |
| 21 | HSPA5 | na | 23780 | -0.053 | -0.3859 | No |
| 22 | IL6 | na | 24766 | -0.067 | -0.3957 | Yes |
| 23 | PRNP | na | 25295 | -0.075 | -0.3902 | Yes |
| 24 | LAMC1 | na | 25472 | -0.078 | -0.3738 | Yes |
| 25 | PRKACB | na | 26730 | -0.102 | -0.3819 | Yes |
| 26 | NCAM2 | na | 27721 | -0.124 | -0.3762 | Yes |
| 27 | PRKACG | na | 28125 | -0.134 | -0.3509 | Yes |
| 28 | IL1B | na | 28762 | -0.150 | -0.3278 | Yes |
| 29 | MAP2K1 | na | 29246 | -0.164 | -0.2965 | Yes |
| 30 | C5 | na | 29749 | -0.181 | -0.2611 | Yes |
| 31 | IL1A | na | 31036 | -0.222 | -0.2372 | Yes |
| 32 | FYN | na | 31175 | -0.227 | -0.1785 | Yes |
| 33 | CCL5 | na | 32225 | -0.277 | -0.1323 | Yes |
| 34 | C1QB | na | 33014 | -0.329 | -0.0643 | Yes |
| 35 | NCAM1 | na | 33867 | -0.412 | 0.0248 | Yes |
Table: GSEA details [plain text format]

  

Fig 2: KEGG\_PRION\_DISEASES      
 Blue-Pink O' Gram in the Space of the Analyzed GeneSet

  

Fig 3: KEGG\_PRION\_DISEASES: Random ES distribution      
 Gene set null distribution of ES for **KEGG\_PRION\_DISEASES**
